# Adherence to Annual Lung Cancer Screening and Rates of Cancer Diagnosis

**DOI:** 10.1001/jamanetworkopen.2025.0942

**Published:** 2025-03-18

**Authors:** Roger Y. Kim, Katharine A. Rendle, Nandita Mitra, Christine Neslund-Dudas, Robert T. Greenlee, Stacey A. Honda, Marilyn M. Schapira, Michael J. Simoff, Jihyoun Jeon, Rafael Meza, Debra P. Ritzwoller, Anil Vachani

**Affiliations:** 1 Division of Pulmonary, Allergy and Critical Care, Department of Medicine, Perelman School of Medicine, University of Pennsylvania, Philadelphia; 2 Department of Family Medicine and Community Health, Perelman School of Medicine, University of Pennsylvania, Philadelphia; 3 Department of Biostatistics, Epidemiology and Informatics, University of Pennsylvania, Philadelphia; 4 Department of Public Health Sciences, Henry Ford Health, Detroit, Michigan; 5 Marshfield Clinic Research Institute, Marshfield, Wisconsin; 6 Center for Health Research, Kaiser Permanente Hawaii, Oahu; 7 Division of General Internal Medicine, Department of Medicine, Perelman School of Medicine, University of Pennsylvania; 8 Department of Pulmonary and Critical Care Medicine, Henry Ford Hospital, Detroit, Michigan; 9 Department of Epidemiology, University of Michigan, Ann Arbor; 10 Department of Integrative Oncology, BC Cancer Research Institute, Vancouver, British Columbia, Canada; 11 School of Population and Public Health, University of British Columbia, Vancouver, British Columbia, Canada; 12 Institute for Health Research, Kaiser Permanente Colorado, Denver

## Abstract

**Importance:**

Adherence to annual lung cancer screening (LCS) is a proposed quality metric for LCS programs, but data linking annual adherence to lung cancer outcomes are lacking.

**Objective:**

To investigate annual LCS adherence rates across 2 subsequent LCS rounds among adults undergoing baseline LCS and examine the association of adherence with lung cancer diagnosis rates.

**Design, Setting, and Participants:**

This retrospective cohort study included adults aged 55 to 75 years who formerly or currently smoked and underwent baseline LCS between January 1, 2015, and December 31, 2018, across 5 US health care systems in the Population-Based Research to Optimize the Screening Process–Lung Consortium. Participants with missing Lung Computed Tomography Screening Reporting & Data System scores or a lung cancer diagnosis prior to LCS initiation were excluded. Data were analyzed from October 2023 to October 2024.

**Exposures:**

For negative baseline screening results, T1 and T2 screening adherence was defined as chest computed tomography (CT) between 10 and 18 months and 22 and 30 months after baseline, respectively. For positive baseline screening results, T1 and T2 adherence was defined as chest CT between 11 and 21 months and 28 and 36 months after baseline, respectively.

**Main Outcomes and Measures:**

The main outcomes were annual T1 and T2 LCS adherence rates and associations between T1 and T2 screening adherence; annual incident lung cancer diagnoses in rounds T0 (0-12 months after baseline), T1 (>12 to 24 months after baseline), and T2 (>24 to 36 months after baseline); and cancer stage distribution.

**Results:**

A total of 10 170 individuals received baseline LCS (median age, 65 years [IQR, 60-69 years]; 5415 [53.2%] male). During round T1, 6141 of 10 033 eligible patients (61.2% [95% CI, 60.2%-62.2%]) were adherent, and during round T2, 5028 of 9966 eligible patients (50.5% [95% CI, 49.5%-51.4%]) were adherent. T1 adherence was significantly associated with T2 adherence (adjusted relative risk, 2.40; 95% CI, 2.06-2.79). Across 36 months of follow-up, 279 patients (2.7%; 95% CI, 2.4%-3.1%) were diagnosed with lung cancer. Incident lung cancer diagnosis rates were 1.3% (95% CI, 1.1%-1.6%), 0.7% (95% CI, 0.5%-0.8%), and 0.8% (95% CI, 0.6%-0.9%) during rounds T0, T1, and T2, respectively. Lung cancer diagnosis rates were higher among individuals who were LCS adherent vs nonadherent during both rounds T1 (59 of 6141 [1.0%; 95% CI, 0.7%-1.2%] vs 8 of 3892 [0.2%; 95% CI, 0.1%-0.4%]; *P* < .001) and T2 (63 of 5028 [1.3%; 95% CI, 1.0%-1.6%] vs 12 of 4938 [0.2%; 95% CI, 0.1%-0.4%]; *P* < .001). A greater proportion of early-stage lung cancers were diagnosed among individuals adherent to screening at T2 compared with those who were not (46 of 63 [73.0%] vs 3 of 12 [25.0%]; *P* = .006).

**Conclusions and Relevance:**

In this multicenter cohort study of adults undergoing LCS, screening adherence was associated with increased overall and early-stage lung cancer detection rates; however, adherence decreased annually after baseline screening, suggesting that it is an important LCS quality metric.

## Key Points

**Question:**

Among adults undergoing lung cancer screening (LCS), what is the adherence to annual screening and is it associated with lung cancer outcomes?

**Findings:**

In this multicenter cohort study of 10 170 adults undergoing LCS, annual screening adherence rates were 61% at year 1 and 51% at year 2. Annual LCS adherence was associated with increased incident lung cancer diagnosis rates during both time frames.

**Meaning:**

In this study, adherence to annual LCS was associated with an increased lung cancer detection rate but decreased annually after baseline screening, suggesting that adherence is an important quality metric for LCS programs.

## Teaser

This cohort study examines adherence to annual lung cancer screening and whether adherence is associated with lung cancer diagnosis rates.

## Introduction

Lung cancer screening (LCS) with low-dose computed tomography (LDCT) reduces lung cancer–specific mortality by increasing the proportion of lung cancers diagnosed at an early stage^1,2,3^ and has been recommended for adults at high risk of lung cancer by the US Preventive Services Task Force since 2013.^4,5^ However, to realize the benefit of LCS in clinical practice, health care systems must establish programs capable of not only identifying and screening eligible adults^6,7^ but also ensuring that screened individuals continue to receive annual LCS.^8,9^ Clinical trial LCS protocols have required annual screening spanning several years, and most lung cancers were detected in follow-up during subsequent rounds of screening.^1,2^ A microsimulation modeling study estimated that the number of lung cancer deaths averted decreased from 501 to 175 per 100 000 persons as annual LCS adherence decreased from 100% to 29%, highlighting the importance of continued screening engagement to achieve lung cancer mortality reduction.^10^

The Lung CT Screening Reporting & Data System (Lung-RADS) provides a framework for LCS recommendations and has been widely adopted in clinical practice.^11^ For negative screening results (Lung-RADS score of 1 or 2), continued annual LCS is recommended, whereas for positive screening results (Lung-RADS score of 3 or 4), short-term imaging follow-up or diagnostic biopsy is recommended. Prior studies assessing LCS adherence have largely been limited to examinations of adherence to Lung-RADS immediate follow-up recommendations after baseline LCS^8,9,12,13,14,15,16,17,18,19,20,21,22,23,24,25^ and have used a heterogenous array of adherence definitions.^26^ At the heart of what makes annual adherence across multiple LCS rounds challenging to measure is the heterogeneity of the follow-up testing and procedures patients undergo when baseline screening results are positive. For example, if a patient has a baseline Lung-RADS screening score of 3, repeat imaging in 6 months is recommended, and assuming a lung cancer diagnosis does not result from this workup, the next LCS round would be 12 months from this follow-up imaging (18 months after baseline LCS). For comparison, in the National Lung Screening Trial (NLST), adherence to subsequent rounds of annual screening was defined as 12-month intervals after baseline screening irrespective of the baseline LCS result,^1^ which is inconsistent with modern Lung-RADS recommendations.^11^ Therefore, efforts to emulate clinical trial definitions of screening adherence are problematic and anachronistic. To date, a standardized method to define and measure annual LCS adherence across multiple LCS rounds has not been established, which hinders LCS programs’ ability to evaluate adherence quality metrics recommended by national societies.^27,28^ Moreover, to our knowledge, data linking annual LCS adherence to lung cancer outcomes do not currently exist. The objectives of this study were to introduce and apply a pragmatic definition of longitudinal annual LCS adherence to estimate annual LCS rates and examine the association between LCS adherence, incident lung cancer detection rates, and cancer stage distribution across 3 rounds of screening in a diverse, community-based multicenter cohort of patients undergoing LCS.

## Methods

### Study Population and Setting

This was a multicenter retrospective cohort study of adults undergoing LCS within the Population-Based Research to Optimize the Screening Process (PROSPR)–Lung Consortium, which comprises 5 US health care systems: Kaiser Permanente Colorado, Kaiser Permanente Hawaii, Henry Ford Health System, Marshfield Clinic Health System, and University of Pennsylvania Health System.^29^ PROSPR-Lung uses a common data model of harmonized patient-level data derived from each system’s electronic health record (EHR), cancer registry, administrative, and claims data. This study was approved by the Kaiser Permanente Colorado institutional review board, and informed consent was waived because of the use of deidentified data. The study followed the Strengthening the Reporting of Observational Studies in Epidemiology (STROBE) reporting guideline. Additional details on the study methods can be found in the eMethods in Supplement 1.

**Figure 1.**
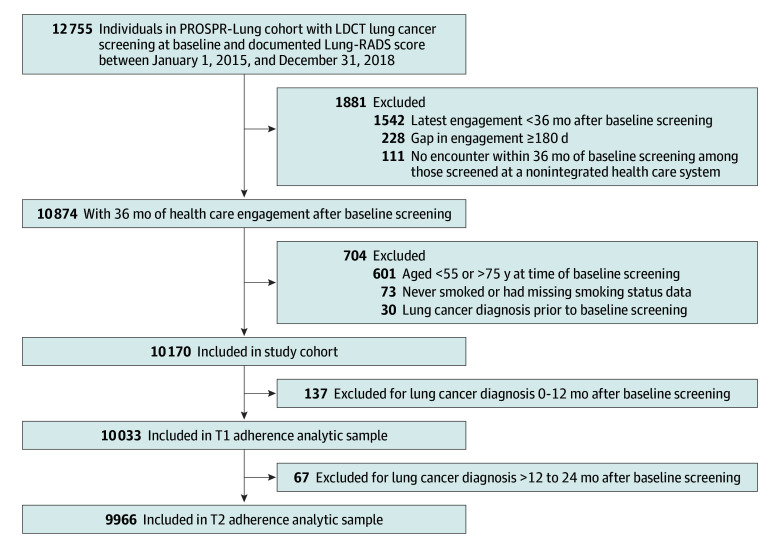
Assembly of the Study Cohort and T1 and T2 Adherence Analytic Samples Among excluded individuals with less than 36 months of health care engagement (n = 1881), 120 (6.4%) were diagnosed with lung cancer and 596 (31.7%) died during the 36-month follow-up period. No individuals in the study cohort died between 0 and 24 months of follow-up after baseline screening. LDCT indicates low-dose computed tomography; Lung-RADS, Lung Computed Tomography Screening Reporting & Data System; PROSPR, Population-Based Research to Optimize the Screening Process; T1, more than 12 months to 24 months after baseline; T2, more than 24 months to 36 months after baseline.

We included individuals aged 55 to 75 years who formerly or currently smoked and underwent baseline LCS with LDCT between January 1, 2015, and December 31, 2018 (Figure 1). All individuals were required to have at least 36 months of follow-up and documented health care engagement after their baseline LCS, allowing us to assess data for 3 rounds of LCS. We excluded individuals with missing baseline Lung-RADS scores and those with a preexisting lung cancer diagnosis. Patients diagnosed with lung cancer within 12 months of the baseline LCS (T0) were excluded from the T1 adherence analytic sample (>12 to 24 months after baseline), as they would no longer be eligible for ongoing annual LCS. Likewise, those diagnosed with lung cancer more than 12 to 24 months after baseline were excluded from the T2 adherence analytic sample (>24 to 36 months after baseline).

### Variables

**Figure 2.**
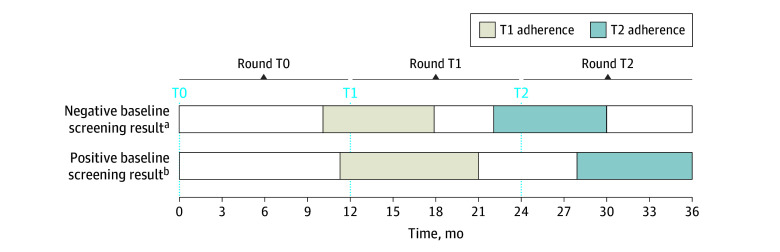
Definitions of Longitudinal Annual Lung Cancer Screening Adherence Round T0 was defined as 0 to 12 months after baseline, round T1 as more than 12 months to 24 months after baseline, and round T2 as more than 24 months to 36 months after baseline. ^a^Among individuals with negative baseline screening results (Lung Computed Tomography Screening Reporting & Data System [Lung-RADS] score of 1 or 2), T1 and T2 adherence was defined as any repeat computed tomography chest scan within 10 to 18 months and 22 to 30 months after baseline, respectively. ^b^Among individuals with positive baseline screening results (Lung-RADS score of 3 or 4), T1 and T2 adherence was defined as any repeat computed tomography chest scan within 11 to 21 months and 28 to 36 months after baseline, respectively.

We collected baseline demographic and clinical data at the baseline LCS. Race and ethnicity information was based on EHR data^30^ and was included in the analysis because our group has previously shown that LCS adherence varies by race.^14^ Categories were American Indian or Alaska Native, Asian, Black, Hispanic, Native Hawaiian or Other Pacific Islander, and White. By Lung-RADS score,^11^ we categorized baseline LCS results as either negative (score of 1 or 2) or positive (score of 3 or 4), as previously described.^14,31,32^ We calculated adherence to annual LCS at T1 and T2 based on the baseline LCS result (Figure 2 and eTable 1 in Supplement 1). Among individuals with negative baseline screening results who were recommended for repeat screening in 12 months, T1 and T2 adherence was defined as any repeat chest CT (ie, not restricted to LDCT) within 10 to 18 months and 22 to 30 months after baseline, respectively. For those with positive baseline screening results, T1 and T2 adherence was defined as any repeat chest CT within 11 to 21 months and 28 to 36 months after baseline, respectively. This phase-shifting of T1 and T2 adherence windows for individuals with positive baseline screening results compared with those with negative baseline screening results accounted for the heterogenous immediate follow-up of screen results that were consistent with a possible suspicion of malignant neoplasm. We allowed for broad adherence windows to account for variability in longitudinal annual LCS as part of routine clinical care (Figure 2).

Based on cancer registry data, we evaluated incident lung cancer diagnoses made during each of the 3 years following the baseline LCS: round T0 (0-12 months after baseline), round T1 (>12 to 24 months after baseline), and round T2 (>24 to 36 months after baseline). We calculated lung cancer diagnosis rates by round of screening and investigated associations between adherence at T1 and T2, incident lung cancer diagnoses, and stage distribution.

### Statistical Analysis

We calculated adherence to annual LCS at T1 and T2 as the proportions of patients not previously diagnosed with lung cancer who received chest CT within the appropriate follow-up time frames based on the baseline LCS result (Figure 2). The rate of incident lung cancer diagnoses was calculated for each LCS round as the number of new lung cancer diagnoses during the specific round divided by the number of individuals eligible for LCS during that round. Using descriptive statistics and the Pearson χ^2^ test, we compared the unadjusted differences in T2 adherence by T1 adherence and differences in T1 and T2 incident lung cancer diagnoses by T1 and T2 adherence, respectively. We used mixed-effects multivariable modified Poisson regression modeling to estimate the adjusted relative risk (ARR) and 95% CI of (1) T2 adherence after T1 adherence (eTable 2 in Supplement 1), (2) T1 lung cancer diagnoses after T1 adherence (eTable 3 in Supplement 1), and (3) T2 lung cancer diagnoses after T1 and T2 adherence (eTable 4 in Supplement 1).^33,34^ Models were adjusted for potential confounders, and health care system was included as a random effect to account for clustering. We used multiple imputation by chained equations to account for missing data.^35^ We performed a sensitivity analysis using NLST definitions for T1 (11- to 15-month chest CT) and T2 (23- to 27-month chest CT) adherence among individuals with negative baseline screening results. All statistical tests were 2-sided, and we considered *P* < .05 to be statistically significant. All analyses were conducted from October 2023 to October 2024 using Stata/MP, version 18.0 (StataCorp LLC).

## Results

Among the 10 170 adults who received baseline LCS between 2015 and 2018 (Table 1), 8592 (84.5%) had negative screening results and 1578 (15.5%) had positive screening results at baseline. The median age was 65 years (IQR, 60-69 years); 4755 patients (46.8%) were identified as female and 5415 (53.2%) as male. A total of 65 (0.6%) were American Indian or Alaska Native; 328 (3.2%), Asian; 1442 (14.2%), Black; 382 (3.8%), Hispanic; 144 (1.4%), Native Hawaiian or Other Pacific Islander; and 7433 (73.1%), White. Most patients currently smoked (5973 [58.7%]), and 6766 (66.5%) had a Charlson Comorbidity Index of 0 or 1.

**Table 1.** Patient Characteristics by Baseline LCS Result

Variable	Participants^a^		
	Total (N = 10 170)	Negative LCS result (n = 8592)	Positive LCS result (n = 1578)
Age, y			
Median (IQR)	65 (60-69)	64 (60-69)	66 (61-70)
55-60	2729 (26.8)	2396 (27.9)	333 (21.1)
61-65	2907 (28.6)	2467 (28.7)	440 (27.9)
66-69	2363 (23.3)	1971 (22.9)	392 (24.8)
70-75	2171 (21.3)	1758 (20.5)	413 (26.2)
Sex			
Female	4755 (46.8)	4017 (46.8)	738 (46.8)
Male	5415 (53.2)	4575 (53.2)	840 (53.2)
Race or ethnicity			
American Indian or Alaska Native	65 (0.6)	52 (0.6)	13 (0.8)
Asian	328 (3.2)	292 (3.4)	36 (2.3)
Black	1442 (14.2)	1246 (14.5)	196 (12.4)
Hispanic	382 (3.8)	320 (3.7)	62 (3.9)
Native Hawaiian or Other Pacific Islander	144 (1.4)	128 (1.5)	16 (1.0)
White	7433 (73.1)	6233 (72.5)	1200 (76.0)
Missing	376 (3.7)	321 (3.7)	55 (3.5)
Baseline Lung-RADS score			
1	2607 (25.6)	2607 (30.3)	NA
2	5985 (58.8)	5985 (69.7)	NA
3	995 (9.8)	NA	995 (63.1)
4A	388 (3.8)	NA	388 (24.6)
4B/4X	195 (1.9)	NA	195 (12.4)
Health care system			
Henry Ford	3404 (33.5)	2951 (34.3)	453 (28.7)
Kaiser Permanente Colorado	3268 (32.1)	2610 (30.4)	658 (41.7)
Kaiser Permanente Hawaii	618 (6.1)	560 (6.5)	58 (3.7)
Marshfield Clinic	893 (8.8)	733 (8.5)	160 (10.1)
University of Pennsylvania	1987 (19.5)	1738 (20.2)	249 (15.8)
Year of baseline screening			
2015	1406 (13.8)	1148 (13.4)	258 (16.3)
2016	2308 (22.7)	1981 (23.1)	327 (20.7)
2017	2989 (29.4)	2542 (29.6)	447 (28.3)
2018	3467 (34.1)	2921 (34.0)	546 (34.6)
Smoking status			
Current	5973 (58.7)	5046 (58.7)	927 (58.7)
Former	4197 (41.3)	3546 (41.3)	651 (41.3)
Charlson Comorbidity Index			
0	3739 (36.8)	3184 (37.1)	555 (35.2)
1	3027 (29.8)	2541 (29.6)	486 (30.8)
≥2	2992 (29.4)	2509 (29.2)	483 (30.6)
Missing	412 (4.1)	358 (4.2)	54 (3.4)
BMI			
≤24.9	2942 (28.9)	2416 (28.1)	526 (33.3)
25.0-29.9	3528 (34.7)	3013 (35.1)	514 (32.6)
≥30	3614 (35.5)	3087 (35.9)	527 (33.4)
Missing	87 (0.9)	76 (0.9)	11 (0.7)
Annual family income, median (IQR), $	71 808 (53 967-95 959)	71 731 (53 920-96 167)	72 383 (54 381-94 219)
Highest level of education			
High school or less	5848 (57.5)	4969 (57.8)	879 (55.7)
Some college or higher	4123 (40.5)	3454 (40.2)	669 (42.4)
Missing	199 (2.0)	169 (2.0)	30 (1.9)
Yost socioeconomic status index quintile			
1 (Lowest)	1643 (16.2)	1388 (16.2)	255 (16.2)
2	1816 (17.9)	1534 (17.9)	282 (17.9)
3	1955 (19.2)	1656 (19.3)	299 (18.9)
4	2196 (21.6)	1848 (21.5)	348 (22.1)
5 (Highest)	2259 (22.2)	1910 (22.2)	349 (22.1)
Missing	301 (3.0)	256 (3.0)	45 (2.9)

Abbreviations: BMI, body mass index (calculated as weight in kilograms divided by height in meters squared); LCS, lung cancer screening; Lung-RADS, Lung Computed Tomography Screening Reporting & Data System; NA, not applicable.

^a^ Data are presented as number (percentage) of participants unless otherwise indicated.

Of the 10 033 individuals eligible for T1 LCS, 6141 (61.2%; 95% CI, 60.2%-62.2%) were adherent during round T1 (eFigure in Supplement 1). Among the 9966 patients eligible for T2 LCS, 5028 (50.5%; 95% CI, 49.5%-51.4%) were adherent during round T2. There was no association between baseline Lung-RADS score and T1 or T2 adherence (eTable 5 in Supplement 1). The sensitivity analysis using NLST definitions of adherence among patients with negative baseline screening results yielded more conservative estimates (eTable 6 in Supplement 1). Individuals who were adherent during round T1 were more likely to be adherent during round T2 compared with those who were not adherent during round T1 (ARR, 2.40; 95% CI, 2.06-2.79) (eTable 2 in Supplement 1).

Over the 36-month follow-up period, 279 of the 10 170 individuals who underwent baseline LCS (2.7%; 95% CI, 2.4%-3.1%) were diagnosed with lung cancer, with a higher proportion diagnosed among those with positive compared with negative baseline screening results (191 of 1578 [12.1%; 95% CI, 10.5%-13.8%] vs 88 of 8592 [1.0%; 95% CI, 0.8%-1.3%]; *P* < .001) (Table 2). During round T0, 137 of the 10 170 screened individuals (1.3%; 95% CI, 1.1%-1.6%) were diagnosed with an incident lung cancer. During round T1, 67 of the remaining 10 033 patients eligible for T1 LCS (0.7%; 95% CI, 0.5%-0.8%) received a lung cancer diagnosis. A greater proportion of patients who were adherent during round T1 were diagnosed with lung cancer (59 of 6141 [1.0%; 95% CI, 0.7%-1.2%]) compared with those who were nonadherent (8 of 3892 [0.2%; 95% CI, 0.1%-0.4%]) (*P* < .001). During round T2, 75 of the 9966 individuals eligible for T2 LCS (0.8%; 95% CI, 0.6%-0.9%) were diagnosed with lung cancer. Similarly, the incident lung cancer diagnosis rate was higher among those who were adherent during round T2 (63 of 5028 [1.3%; 95% CI, 1.0%-1.6%]) compared with those who were nonadherent (12 of 4938 [0.2%; 95% CI, 0.1%-0.4%]) (*P* < .001). In multivariable analyses, the associations persisted between screening adherence and lung cancer diagnosis during rounds T1 (ARR, 4.64; 95% CI, 2.57-8.37) (eTable 3 in Supplement 1) and T2 (ARR, 5.90; 95% CI, 3.34-10.43) (eTable 4 in Supplement 1) .

**Table 2.** Lung Cancer Diagnosis Rates Across 3 Rounds of Screening

Screening round^a^	Overall^b^		Negative baseline screening result^c^		Positive baseline screening result^d^	
	Total, No.	Lung cancer diagnosis, No. (% [95% CI])	Total, No.	Lung cancer diagnosis, No. (% [95% CI])	Total, No.	Lung cancer diagnosis, No. (% [95% CI])
Overall	10 170	279 (2.7 [2.4-3.1])	8592	88 (1.0 [0.8-1.3])	1578	191 (12.1 [10.5-13.8])
T0	10 170	137 (1.3 [1.1-1.6])	8592	7 (0.1 [0.0-0.2])	1578	130 (8.2 [6.9-9.7])
T1						
All	10 033	67 (0.7 [0.5-0.8])	8585	34 (0.4 [0.3-0.6])	1448	33 (2.3 [1.6-3.2 ])
Adherent	6141	59 (1.0 [0.7-1.2])	5247	28 (0.5 [0.4-0.8])	894	31 (3.5 [2.4-4.9])
Nonadherent	3892	8 (0.2 [0.1-0.4])	3338	6 (0.2 [0.1-0.4])	554	2 (0.4 [0.0-1.3])
T2						
All	9966	75 (0.8 [0.6-0.9])	8551	47 (0.6 [0.4-0.7])	1415	28 (2.0 [1.3-2.8])
Adherent	5028	63 (1.3 [1.0-1.6])	4358	36 (0.8 [0.6-1.1])	670	27 (4.0 [2.7-5.8])
Nonadherent	4938	12 (0.2 [0.1-0.4])	4193	11 (0.3 [0.1-0.5])	745	1 (0.1 [0.0-0.7])

^a^ Round T0 was defined as 0 to 12 months after baseline, round T1 as more than 12 months to 24 months after baseline, and round T2 as more than 24 months to 36 months after baseline.

^b^ Overall totals are for all individuals across all 3 rounds of screening.

^c^ Lung Computed Tomography Screening Reporting & Data System score of 1 or 2.

^d^ Lung Computed Tomography Screening Reporting & Data System score of 3 or 4.

Notably, T1 adherence was not associated with lung cancer diagnosis during round T2 (ARR, 0.86; 95% CI, 0.60-1.23) (eTable 4 in Supplement 1). This finding was largely attributable to the high lung cancer diagnosis rate among individuals who were nonadherent during round T1 but were adherent during round T2. As shown in the eFigure in Supplement 1, of 6082 patients with T1 adherence who were not diagnosed with cancer during round T1, 4072 (67.0%; 95% CI, 65.8%-68.1%) were adherent during round T2. In comparison, 956 of the 3884 T2-eligible patients who were nonadherent during round T1 completed a T2 screening (24.6%; 95% CI, 23.3%-26.0%), and 20 of these patients (2.1%; 95% CI, 1.3%-3.2%) were diagnosed with lung cancer during round T2. Among the 2928 individuals who were nonadherent during both rounds T1 and T2, only 4 (0.1%; 95% CI, 0.04%-0.3%) were diagnosed with lung cancer during round T2.

Of the 279 total lung cancer diagnoses within 36 months of baseline LCS, 204 (73.1%; 95% CI, 67.5%-78.2%) represented early-stage disease (stage 0, I, or II); 38 (13.6%; 95% CI, 9.8%-18.2%), regional disease (stage III); and 24 (8.6%; 95% CI, 5.6%-12.5%), distant disease (stage IV) (Table 3). Lung cancer stage distribution during round T1 was not significantly different by LCS adherence. However, T2-adherent individuals diagnosed with lung cancer during round T2 were more likely to have early-stage (0, I, or II) disease (46 of 63 [73.0%] vs 3 of 12 [25.0%]) and less likely to have late-stage (III or IV) disease (13 of 63 [20.6%] vs 7 of 12 [58.3%]) (*P* = .006) compared with those nonadherent during round T2 (Table 3 and eTable 7 in Supplement 1).

**Table 3.** Lung Cancer Diagnoses by Round of Screening, T1 and T2 Adherence, and Stage at Diagnosis

Stage at diagnosis	Lung cancer diagnoses, No. (% [95% CI])^a^					
	Overall (N = 279)^b^	Round T0 (n = 137)	Round T1 (n = 67)		Round T2 (n = 75)	
			Adherent (n = 59)	Nonadherent (n = 8)	Adherent (n = 63)	Nonadherent (n = 12)
0	2 (0.7 [0.1-2.6])	0	1 (1.7 [0.0-9.1])	0	0	1 (8.3 [0.2-38.5])
I	179 (64.2 [58.2-69.8])	87 (63.5 [54.9-71.6])	43 (72.9 [59.7-83.6])	7 (87.5 [47.3-99.7])	41 (65.1 [52.0-76.7])	1 (8.3 [0.2-38.5])
II	23 (8.2 [5.3-12.1])	13 (9.5 [5.1-15.7])	4 (6.8 [1.9-16.5])	0	5 (7.9 [2.6-17.6])	1 (8.3 [0.2-38.5])
III	38 (13.6 [9.8-18.2])	22 (16.1 [10.3-23.3])	6 (10.2 [3.8-20.8])	0	7 (11.1 [4.6-21.6])	3 (25.0 [5.5-57.2])
IV	24 (8.6 [5.6-12.5])	10 (7.3 [3.6-13.0])	3 (5.1 [1.1-14.1])	1 (12.5 [0.3-52.7])	6 (9.5 [3.6-19.6])	4 (33.3 [9.9-65.1])
Missing	13 (4.7 [2.5-7.8])	5 (3.7 [1.2-8.3])	2 (3.4 [0.4-11.7])	0	4 (6.4 [1.8-15.5])	2 (16.7 [2.1-48.4])

^a^ Round T0 was defined as 0 to 12 months after baseline, round T1 as more than 12 months to 24 months after baseline, and round T2 as more than 24 months to 36 months after baseline.

^b^ Overall totals are for all individuals across all 3 rounds of screening.

## Discussion

In this multicenter cohort study of adults who received baseline LCS between 2015 and 2018 across 5 US health care systems, adherence to annual LCS decreased with each round of screening, and adherence during round T1 was associated with subsequent round T2 adherence. Annual LCS adherence was significantly associated with increased lung cancer detection during each round of screening and a greater ratio of early- to late-stage disease by round T2. To our knowledge, this is the first study to demonstrate that annual LCS adherence was associated with clinically meaningful improvements in early detection of lung cancer and favorable stage distribution.

We developed and evaluated a pragmatic definition of annual LCS adherence based solely on individuals’ baseline Lung-RADS score, categorized as either negative (Lung-RADS 1 or 2) or positive (Lung-RADS 3 or 4) and measured by receipt of any chest CT during clinically relevant time intervals. This simplified approach directly accounts for the heterogeneity of immediate diagnostic testing and procedures often following positive baseline LCS results (ie, those in which a non–benign-appearing pulmonary nodule is detected) by phase-shifting the appropriate time frame for subsequent annual LCS rounds back a few months. By capturing any chest CT scans performed during these broad time windows for adherence, our definitions for longitudinal annual LCS adherence reflect the realities of LCS in routine clinical care. For example, a patient with a Lung-RADS score of 2 at baseline screening who is recommended to return in 12 months for a T1 LDCT screening may instead receive a diagnostic chest CT scan at 16 months as part of an evaluation for an acute respiratory tract infection in the emergency department. Since this diagnostic CT scan would still be able to provide the clinical information necessary for annual LCS (ie, presence or change in size of a pulmonary nodule), it makes practical sense to consider this imaging study as T1 adherence. This practical, updated approach to calculating annual LCS adherence helps to explain why our estimates of adherence are higher than those previously reported by our group^14,31^ and others.^12,13,17,36,37^

T1 adherence was associated with T2 adherence, with 67.0% of those adherent during round T1 also adherent during round T2 compared with 24.6% of those nonadherent during round T1. Although most individuals who did not receive a round T1 screening also missed a T2 screening, among those who did receive T2 LCS, the incident lung cancer detection rate was 2.1%. Additionally, adherence during round T1 was not independently associated with an increased incident lung cancer diagnosis rate during round T2, emphasizing the importance of each round of annual LCS after baseline screening. These results suggest that if LCS programs were to proactively identify eligible individuals who miss T1 screening to reengage them for round T2 screening, they could make a meaningful impact on these patients’ clinical trajectory by detecting more early-stage cancers and potentially averting downstream lung cancer–related deaths. Moreover, these findings suggest that our pragmatic definitions of adherence capture clinically relevant information that directly corresponds to patient lung cancer outcomes.

### Strengths and Limitations

The primary strength of our analysis is its focus on the association between annual LCS adherence and lung cancer diagnosis outcomes. Most prior analyses of LCS adherence only assessed 1 follow-up round of screening among individuals with negative baseline screening results or the immediate diagnostic evaluation after baseline screening among those with positive baseline screening results.^12,13,14,17,25,31^ Our study is distinct in that it assessed multiple rounds of screening, accounting for the heterogeneity in diagnostic evaluation that often occurs after a positive baseline screening result. Previous studies assessing annual LCS adherence across multiple rounds of screening have not directly assessed lung cancer diagnosis rates or stage distribution.^37,38,39^ Thus, to our knowledge, this is the first study to find the association of annual LCS adherence with patient outcomes suggested by prior microsimulation modeling.^10^ Another strength of this analysis is the generalizability of its findings. The study population represented a large and diverse cohort of adults undergoing LCS as part of routine clinical care. Notably, the cohort included individuals screened at 5 community-based US health care systems with both centralized and decentralized LCS programs, suggesting that our results may apply to a variety of clinical settings. Additionally, the data model used in this analysis integrated individual-level information from EHR, cancer registry, administrative, and claims data sources, allowing us to capture all clinical encounters and minimize the likelihood of misclassification bias. The routine and easily accessible variables included in our analysis also suggest that other LCS programs could similarly implement our annual LCS adherence definitions as quality metrics. From an analytical perspective, another strength of our study was that each individual had 36 months of follow-up data available, eliminating the possibility of variable follow-up duration and allowing us to assess LCS across 3 rounds.

Our study also has limitations. First, as this was an observational study, the possibility of unmeasured confounding influencing our results exists. However, we adjusted for several clinically relevant measured confounders in multivariable modeling, and the magnitude of our reported adjusted effect estimates suggests that any unmeasured confounding would be unlikely to completely account for the associations between annual T1 and T2 LCS adherence and incident lung cancer diagnosis. Second, the risk of detection bias cannot be completely excluded, as the dataset was restricted to data from the 5 participating health care systems. Thus, it is theoretically possible that individuals could have received LCS-related care or lung cancer diagnoses outside the PROSPR-Lung Consortium. However, we strived to mitigate this risk by excluding individuals who did not have 36 months of health care engagement and a documented health care encounter at participating sites during follow-up after baseline LCS. In doing so, we restricted our analysis to individuals who were both alive and had insurance coverage throughout the follow-up period. Therefore, estimates of incident lung cancer diagnoses reported in this study did not include individuals who lacked 36 months of health care engagement after their baseline LCS. Third, we excluded individuals who were diagnosed with lung cancer or who died during previous rounds of screening from subsequent analyses of annual adherence, as they would no longer be eligible for ongoing screening. Additionally, we chose not to use marginal structural modeling for our analytic approach. This strategy could theoretically lead to biased results if attempting to make causal inferences^40^; however, the goal of this study was not to imply causation or a mechanistic explanation but instead simply to investigate associations between screening adherence and lung cancer detection. Fourth, due to incomplete smoking history data for quit date among individuals who formerly smoked and a lack of shared decision-making documentation available in our dataset, it is possible that some patients may have become ineligible for ongoing LCS during subsequent rounds of screening.^41^

## Conclusions

In this cohort study, use of a pragmatic definition of annual LCS adherence was associated with increased incident lung cancer diagnosis rates and a favorable stage distribution among patients undergoing routine LCS. The finding that annual LCS adherence rates decreased across subsequent rounds of screening supports the use of annual adherence as a quality metric for LCS programs seeking to maximize the benefits of LCS for early lung cancer detection and, ultimately, reduced lung cancer-related deaths.

## Notes

Supplement 1.
eMethods.
**eTable 1.** Lung-RADS Category Descriptors and Study Definitions of Annual Adherence **eTable 2.** Multivariable Model for Factors Associated With T2 Adherence **eTable 3.** Multivariable Model for Factors Associated with Incident Lung Cancer Diagnosis During Round T1 **eTable 4.** Multivariable Model for Factors Associated With Incident Lung Cancer Diagnosis During Round T2 **eTable 5.** T1 and T2 Adherence Stratified by Baseline Lung-RADS Score **eTable 6.** Sensitivity Analysis Using NLST Definitions for T1 and T2 Adherence Among Individuals With Negative Baseline Screen Test Results **eTable 7.** Stage Distribution of Lung Cancers Diagnosed During Round T2, Stratified by T1 and T2 Adherence **eFigure.** Flowchart of Study Participant Longitudinal Lung Cancer Screening Adherence and Incident Lung Cancer Diagnoses Across 3 Rounds of Screening
eReferences.

Supplement 2.
Data Sharing Statement
