# Supplementary material for: Adherence to Annual Lung Cancer Screening and Rates of Cancer Diagnosis
Source: JAMA Netw Open. Author manuscript; Available in PMC 2025 Apr 12. (PMC11920840; doi:10.1001/jamanetworkopen.2025.0942)
Supplement: Supplement 1 — eMethods. eTable 1. Lung-RADS Category Descriptors and Study Definitions of Annual Adherence eTable 2. Multivariable Model for Factors Associated With T2 Adherence eTable 3. Multivariable Model for Factors Associated with Incident Lung Cancer Diagnosis During Round T1 eTable 4. Multivariable Model for Factors Associated With Incident Lung Cancer Diagnosis During Round T2 eTable 5. T1 and T2 Adherence Stratified by Baseline Lung-RADS Score eTable 6. Sensitivity Analysis Using NLST Definitions for T1 and T2 Adherence Among Individuals With Negative Baseline Screen Test Results eTable 7. Stage Distribution of Lung Cancers Diagnosed During Round T2, Stratified by T1 and T2 Adherence eFigure. Flowchart of Study Participant Longitudinal Lung Cancer Screening Adherence and Incident Lung Cancer Diagnoses Across 3 Rounds of Screening eReferences. [file NIHMS2067533-supplement-Supplement_1.pdf]

Supplementary Online Content

Kim RY, Rendle KA, Mitra N, et al. Adherence to annual lung cancer screening and rates of cancer diagnosis. *JAMA Netw Open*. 2025;8(3):e250942.
doi:10.1001/jamanetworkopen.2025.0942

eMethods.
eTable 1. Lung-RADS Category Descriptors and Study Definitions of Annual Adherence
eTable 2. Multivariable Model for Factors Associated With T2 Adherence
eTable 3. Multivariable Model for Factors Associated with Incident Lung Cancer Diagnosis During Round T1
eTable 4. Multivariable Model for Factors Associated With Incident Lung Cancer Diagnosis During Round T2
eTable 5. T1 and T2 Adherence Stratified by Baseline Lung-RADS Score
eTable 6. Sensitivity Analysis Using NLST Definitions for T1 and T2 Adherence Among Individuals With Negative Baseline Screen Test Results
eTable 7. Stage Distribution of Lung Cancers Diagnosed During Round T2, Stratified by T1 and T2 Adherence
eFigure. Flowchart of Study Participant Longitudinal Lung Cancer Screening Adherence and Incident Lung Cancer Diagnoses Across 3 Rounds of Screening
eReferences.

This supplementary material has been provided by the authors to give readers additional information about their work.

© 2025 Kim RY et al. *JAMA Network Open*.

eMethods.

Study Population and Setting

This was a multicenter retrospective cohort study of adults undergoing LCS within the Population-based Research to Optimize the Screening Process (PROSPR) Lung Consortium, which comprises five U.S. healthcare systems: Kaiser Permanente Colorado, Kaiser Permanente Hawaii, Henry Ford Health System, Marshfield Clinic Health System, and University of Pennsylvania Health System.[1] The PROSPR Lung Consortium is a National Cancer Institute-funded collaboration focused on optimizing LCS in clinical practice and uses a common data model of harmonized patient-level data derived from each healthcare system's electronic health record (EHR), cancer registry, administrative, and claims data. This study adheres to the Strengthening the Reporting of Observational Studies in Epidemiology reporting guidelines[2] and was approved by the Kaiser Permanente Colorado Institutional Review Board.

Assembly of the study cohort is summarized in **Figure 1**. As LCS is a covered Medicare benefit through age 77, we included individuals aged 55 to 75 years who formerly or currently smoked and underwent baseline (T0) LCS with LDCT (*Healthcare Common Procedure Coding System* codes G0297 and S8032) between January 1, 2015, and December 31, 2018. All individuals were required to have at least 36 months of follow-up and documented healthcare engagement after T0 LCS (i.e., follow-up through December 31, 2021). Using information on active insurance coverage on file at each PROSPR Lung healthcare system, we ensured that each individual had documented engagement from the T0 screen until at least 36 months after without any breaks in engagement greater than 180 days. Additionally, among individuals screened at non-integrated health systems (i.e., University of Pennsylvania, Marshfield Clinic, and Henry Ford), we required a minimum of one documented healthcare encounter over the 36-month follow-up period. This allowed us to assess data for three rounds of LCS: T0 (0 to 12 months), T1 (>12 to 24 months), and T2 (>24 to 36 months). We excluded individuals with missing T0 Lung-RADS scores and those with preexisting lung cancer diagnoses. Patients diagnosed with lung cancer within 12 months of T0 LCS were excluded from the T1 adherence analytic sample, as they would no longer be eligible for ongoing annual LCS. Likewise, those diagnosed with lung cancer >12 to 24 months were excluded from the T2 adherence analytic sample (**Figure 1**).

Variables

For each study participant, we collected baseline data at the time of T0 LCS on demographics, Lung-RADS score, healthcare system, year of T0 LCS, smoking status, Charlson Comorbidity Index (CCI),[3] body mass index (BMI), and census tract-based socioeconomic status (median family income, highest education level attained, and Yost index quintile[4]). Race and ethnicity information was based on EHR data.[5] By Lung-RADS score,[6] we categorized T0 LCS results as either negative (Lung-RADS 1 or 2) or positive (Lung-RADS 3 or 4), as previously described.[7-9]

We calculated adherence to annual LCS at T1 and T2 based on T0 LCS result and receipt of CT chest imaging based on CPT, ICD-9, and ICD-10 procedural codes (**Figure 2, eTable 1**). Among individuals with negative T0 screens recommended for repeat screening in 12 months, T1 and T2 adherence was defined as any repeat CT chest scan (i.e., not restricted to LDCT) within 10-18 months and 22-30 months after T0, respectively. For those with positive T0 screens, T1 and T2 adherence was defined as any repeat CT chest scan within 11-21 months and 28-36 months after T0, respectively. This phase-shifting of T1 and T2 adherence windows for individuals with positive T0 screens relative to those with negative screens accounted for the heterogeneous immediate follow-up of abnormal T0 LCS findings. While a patient with a Lung-RADS 3 T0 screen should undergo follow-up imaging at 6 months, pushing the typical 12-month recommended round T1 timepoint back to 18 months after T0, a patient with a Lung-RADS 4A T0 screen might undergo a 3-month CT chest or positron emission tomography/CT scan as part of immediate work-up, which would reset the T1 timepoint to 13 to 15 months after T0. We allowed for broad adherence windows to account for variability in longitudinal annual LCS as part of routine clinical care (**Figure 2**). We additionally performed a sensitivity analysis using NLST definitions for T1 (11 to 15-month CT chest) and T2 (23 to 27-month CT chest) adherence among individuals with negative T0 screens.

Based on cancer registry data, we evaluated incident lung cancer diagnoses made during each of the three years following T0 LCS: round T0 (0 to 12 months after T0), round T1 (>12 to 24 months after T0), and round T2 (>24 to 36 months after T0). For lung cancer diagnoses from 2015-2017, the American Joint Committee on Cancer (AJCC) 7th Edition was used for staging, and for those from 2018-2021, the AJCC 8th Edition was used. We calculated lung cancer diagnosis rates by round of screening and investigated associations between adherence at T1 and T2, incident lung cancer diagnoses, and stage distribution at diagnosis.

© 2025 Kim RY et al. *JAMA Network Open*.

Statistical Analysis

We used descriptive statistics to assess and report patient demographics and clinical characteristics. Medians and interquartile ranges (IQR) were used to describe continuous variables, and frequencies and percentages for categorical variables. We calculated adherence to annual LCS at T1 and T2 as the proportions of patients not previously diagnosed with lung cancer who received repeat CT chest scans within the appropriate follow-up timeframes based on T0 LCS result (**Figure 2**). The rate of incident lung cancer diagnoses was calculated for each LCS round as the number of new lung cancer diagnoses during the specific round, divided by the number of individuals eligible for LCS during that round. **Figure 2** outlines the specific time interval for each round.

Using descriptive statistics and the Pearson's chi-squared test, we compared the unadjusted differences in T2 adherence by T1 adherence and differences in T1 and T2 incident lung cancer diagnoses by T1 and T2 adherence, respectively. We then used mixed-effects multivariable modified Poisson regression modeling to estimate the adjusted relative risks (aRR) and 95% confidence intervals (CI) of 1) T1 adherence for T2 adherence (**eTable 2**), 2) T1 adherence for T1 lung cancer diagnoses (**eTable 3**), and 3) T1 and T2 adherence for T2 lung cancer diagnoses (**eTable 4**).[10,11] All models were adjusted for T0 Lung-RADS score, age, sex, race, smoking status, year of T0 LCS, CCI, BMI, and Yost index quintile, and healthcare system was included as a random effect to account for clustering. We imputed missing values for race (3.7%), CCI (4.1%), BMI (0.9%), and Yost index quintile (3.0%) using multiple imputation by chained equations.[12] For each model, we generated 10 multiply imputed datasets based on the original dataset, which were combined using Rubin's rules.[13] The regression models were then fit on each imputed dataset and summarized across datasets using Rubin's rules.[12]

All statistical tests were two-sided, and we considered a $P<0.05$ to be statistically significant. All analyses were conducted using Stata/MP 18.0.

© 2025 Kim RY et al. *JAMA Network Open*.

eTable 1. Lung-RADS Category Descriptors and Study Definitions of Annual Adherence.

<table>
<tr><th rowspan="2">Lung-RADS score</th><th rowspan="2">Category Descriptor</th><th rowspan="2">Lung-RADS Recommendation</th><th colspan="2">Study Definitions of Annual Adherence</th></tr>
<tr><th>T1</th><th>T2</th></tr>
<tr><td colspan="2">Negative baseline screen</td><td rowspan="3">12 mo LDCT</td><td rowspan="3">10-18 mo any chest CT*</td><td rowspan="3">22-30 mo any chest CT*</td></tr>
<tr><td>1</td><td>Negative</td></tr>
<tr><td>2</td><td>Benign</td></tr>
<tr><td colspan="3">Positive baseline screen</td><td rowspan="5">11-21 mo any chest CT*</td><td rowspan="5">28-36 mo any chest CT*</td></tr>
<tr><td>3</td><td>Probably benign</td><td>6 mo LDCT</td></tr>
<tr><td>4A</td><td>Suspicious</td><td>3 mo LDCT; PET/CT if ≥8mm solid component</td></tr>
<tr><td>4B</td><td rowspan="2">Very suspicious</td><td rowspan="2">Chest CT, PET/CT, and/or biopsy</td></tr>
<tr><td>4X</td></tr>
</table>

Abbreviations: Lung-RADS=Lung Imaging Reporting and Data System; LDCT=low-dose computed tomography; CT=computed tomography; PET/CT=positron emission tomography/computed tomography

*Included LDCT, chest CT with or without intravenous contrast, and chest CT angiography

© 2025 Kim RY et al. *JAMA Network Open*.

eTable 2. Multivariable Model for Factors Associated with T2 Adherence.

Variable	aRR (95% CI)	*P*-value
**T1 adherence**		
Nonadherent	1.00 (reference)	
**Adherent**	**2.40 (2.06-2.79)**	**<0.001**
Baseline Lung-RADS score		
1	1.00 (reference)	
2	1.03 (0.99-1.07)	0.186
3	0.95 (0.89-1.02)	0.145
4A	0.87 (0.73-1.04)	0.122
4B/4X	1.00 (0.64-1.56)	0.988
Age, years		
55-60	1.00 (reference)	
61-65	1.02 (0.97-1.08)	0.437
66-69	1.07 (0.97-1.18)	0.157
70-75	1.05 (1.01-1.09)	0.011
Sex		
Female	1.00 (reference)	
Male	0.97 (0.94-1.00)	0.095
Race		
White	1.00 (reference)	
Black	1.03 (0.94-1.12)	0.556
Other	0.99 (0.96-1.01)	0.241
Smoking status		
Current	1.00 (reference)	
Former	1.05 (1.00-1.10)	0.054
Year of baseline screening		
2015	1.00 (reference)	
2016	0.99 (0.99-1.09)	0.881
2017	0.98 (0.92-1.05)	0.583
2018	0.95 (0.85-1.06)	0.350
Charlson Comorbidity Index		
0	1.00 (reference)	
1	1.03 (0.99-1.08)	0.144
≥2	1.05 (0.98-1.12)	0.173
Body mass index, kg/m^2		
≤24.9	1.00 (reference)	
25.0-29.9	1.02 (0.94-1.10)	0.643
≥30	1.03 (0.98-1.08)	0.289
Yost socioeconomic status index quintile		
1 (lowest)	1.00 (reference)	
2	1.04 (0.96-1.13)	0.353
3	1.04 (0.92-1.17)	0.534
4	1.05 (0.94-1.16)	0.385
5 (highest)	1.05 (0.93-1.17)	0.434

Abbreviations: aRR=adjusted relative risk; CI=confidence interval; Lung-RADS=Lung Imaging Reporting and Data System

Results of exponentiated beta coefficients for all independent variables in multivariable modified Poisson regression (log-link) model are displayed. Study site heterogeneity was modeled as random effects. Multiple imputation with chained equations was used to account for missing data for race (3.7%), Charlson Comorbidity Index (4.1%), body mass index (0.9%), and Yost index quintile (3.0%).

© 2025 Kim RY et al. *JAMA Network Open*.

eTable 3. Multivariable Model for Factors Associated with Incident Lung Cancer Diagnosis during Round T1 (>12 to 24 Months after T0 Screen).

Variable	aRR (95% CI)	*P*-value
**T1 adherence**		
Nonadherent	1.00 (reference)	
**Adherent**	**4.64 (2.57-8.37)**	**<0.001**
Baseline Lung-RADS score		
1	1.00 (reference)	
2	1.40 (0.26-7.62)	0.694
3	5.15 (1.23-21.59)	0.025
4A	10.84 (2.12-55.50)	0.004
4B/4X	13.71 (2.85-65.83)	0.001
Age, years		
55-60	1.00 (reference)	
61-65	1.25 (0.54-2.86)	0.602
66-69	2.47 (1.16-5.25)	0.019
70-75	1.53 (0.77-3.04)	0.229
Sex		
Female	1.00 (reference)	
Male	0.54 (0.37-0.79)	0.002
Race		
White	1.00 (reference)	
Black	0.57 (0.42-0.77)	<0.001
Other	0.93 (0.27-3.19)	0.912
Smoking status		
Current	1.00 (reference)	
Former	0.68 (0.50-0.91)	0.009
Year of baseline screening		
2015	1.00 (reference)	
2016	1.69 (0.75-3.82)	0.296
2017	1.41 (0.79-2.51)	0.243
2018	1.66 (0.55-4.97)	0.366
Charlson Comorbidity Index		
0	1.00 (reference)	
1	1.06 (0.75-1.50)	0.726
≥2	1.61 (1.01-2.57)	0.047
Body mass index, kg/m^2		
≤24.9	1.00 (reference)	
25.0-29.9	1.08 (0.63-1.84)	0.791
≥30	0.64 (0.31-1.34)	0.236
Yost socioeconomic status index quintile		
1 (lowest)	1.00 (reference)	
2	1.06 (0.32-3.53)	0.923
3	0.63 (0.43-0.92)	0.019
4	0.67 (0.26-1.69)	0.395
5 (highest)	0.28 (0.09-0.82)	0.020

Abbreviations: aRR=adjusted relative risk; CI=confidence interval; Lung-RADS=Lung Imaging Reporting and Data System

Results of exponentiated beta coefficients for all independent variables in multivariable modified Poisson regression (log-link) model are displayed. Study site heterogeneity was modeled as random effects. Multiple imputation with chained equations was used to account for missing data for race (3.7%), Charlson Comorbidity Index (4.1%), body mass index (0.9%), and Yost index quintile (3.0%).

© 2025 Kim RY et al. *JAMA Network Open*.

eTable 4. Multivariable Model for Factors Associated with Incident Lung Cancer Diagnosis during Round T2 (>24 to 36 Months after T0 Screen).

Variable	aRR (95% CI)	*P*-value
**T1 adherence**		
Nonadherent	1.00 (reference)	
**Adherent**	**0.86 (0.60-1.23)**	**0.409**
**T2 adherence**		
Non-adherent	1.00 (reference)	
**Adherent**	**5.90 (3.34-10.43)**	**<0.001**
Baseline Lung-RADS score		
1	1.00 (reference)	
2	1.30 (0.94-1.79)	0.110
3	4.15 (2.13-8.10)	<0.001
4A	4.46 (1.17-16.93)	0.028
4B/4X	7.95 (2.16-29.29)	0.002
Age, years		
55-60	1.00 (reference)	
61-65	0.91 (0.50-1.67)	0.768
66-69	1.24 (0.51-3.03)	0.639
70-75	1.83 (0.69-4.87)	0.226
Sex		
Female	1.00 (reference)	
Male	1.02 (0.67-1.55)	0.935
Race		
White	1.00 (reference)	
Black	1.20 (0.66-2.17)	0.547
Other	0.92 (0.69-1.25)	0.604
Smoking status		
Current	1.00 (reference)	
Former	0.54 (0.39-0.75)	<0.001
Year of baseline screening		
2015	1.00 (reference)	
2016	0.61 (0.42-0.91)	0.014
2017	0.58 (0.49-0.69)	<0.001
2018	0.81 (0.47-1.40)	0.449
Charlson Comorbidity Index		
0	1.00 (reference)	
1	1.10 (0.77-1.57)	0.591
≥2	0.95 (0.47-1.94)	0.894
Body mass index, kg/m^2		
≤24.9	1.00 (reference)	
25.0-29.9	1.46 (0.94-2.27)	0.090
≥30	1.50 (0.91-2.47)	0.110
Yost socioeconomic status index quintile		
1 (lowest)	1.00 (reference)	
2	1.14 (0.63-2.07)	0.662
3	1.08 (0.59-1.97)	0.805
4	0.82 (0.31-2.20)	0.696
5 (highest)	1.19 (0.53-2.64)	0.672

Abbreviations: aRR=adjusted relative risk; CI=confidence interval; Lung-RADS=Lung Imaging Reporting and Data System

Results of exponentiated beta coefficients for all independent variables in multivariable modified Poisson regression (log-link) model are displayed. Study site heterogeneity was modeled as random effects. Multiple imputation with chained equations was used to account for missing data for race (3.7%), Charlson Comorbidity Index (4.1%), body mass index (0.9%), and Yost index quintile (3.0%).

© 2025 Kim RY et al. *JAMA Network Open*.

eTable 5. T1 and T2 Adherence Stratified by Baseline Lung-RADS Score.

Screening Round	Baseline Lung-RADS Score				
	1	2	3	4A	4B/4X
T1	55.6 (1,448/2,606)	63.5 (3,799/5,979)	61.3 (604/985)	66.5 (236/ 355)	50.0 (54/108)
T2	46.9 (1,219/2,599)	52.7 (3,139/5,952)	48.7 (472/969)	44.4 (152/342)	44.2 (46/104)

Abbreviation: Lung-RADS=Lung Imaging Reporting and Data System

Adherence is displayed as percentage adherent (number of individuals/total number). For Lung-RADS 1 or 2 baseline screens, T1 and T2 adherence was defined as repeat CT chest imaging between 10-18 months and 22-30 months, respectively. For Lung-RADS 3, 4A, or 4B/4X baseline screens, T1 and T2 adherence was defined as repeat CT chest imaging between 11-21 months and 28-36 months, respectively.

© 2025 Kim RY et al. *JAMA Network Open*.

eTable 6. Sensitivity Analysis Using NLST Definitions for T1 and T2 Adherence among Individuals with Negative Baseline Screen Test Results (Lung-RADS 1 or 2).

Screening Round	Baseline Lung-RADS Score	
	1	2
T1	46.4 (1,209/2,606)	53.9 (3,220/5,979)
T2	32.7 (849/2,599)	36.7 (2,186/5,952)

Abbreviations: NLST=National Lung Screening Trial; Lung-RADS=Lung Imaging Reporting and Data System

Adherence is displayed as percentage adherent (number of individuals/total number). For both Lung-RADS 1 and 2 baseline screens, T1 and T2 adherence was defined as repeat CT chest imaging between 11-15 months and 23-27 months, respectively.

© 2025 Kim RY et al. *JAMA Network Open*.

eTable 7. Stage Distribution of Lung Cancers Diagnosed during Round T2, Stratified by T1 and T2 Adherence.

Stage at Diagnosis	T1 Adherent		T1 Nonadherent	
	T2 Adherent (*n*=43)	T2 Nonadherent (*n*=8)	T2 Adherent (*n*=20)	T2 Nonadherent (*n*=4)
0	0 (0.0)	1 (12.5)	0 (0.0)	0 (0.0)
I	29 (67.4)	0 (0.0)	12 (60.0)	1 (25.0)
II	3 (7.0)	1 (12.5)	2 (10.0)	0 (0.0)
III	6 (14.0)	2 (25.0)	1 (5.0)	1 (25.0)
IV	2 (4.7)	2 (25.0)	4 (20.0)	2 (50.0)
Missing	3 (7.0)	2 (25.0)	1 (5.0)	0 (0.0)

Incident lung cancer diagnoses by stage at diagnosis are displayed as No. (%).

© 2025 Kim RY et al. *JAMA Network Open*.

eFigure. Flowchart of study participant longitudinal lung cancer screening adherence and incident lung cancer diagnoses across three rounds of screening (T0, T1, T2).

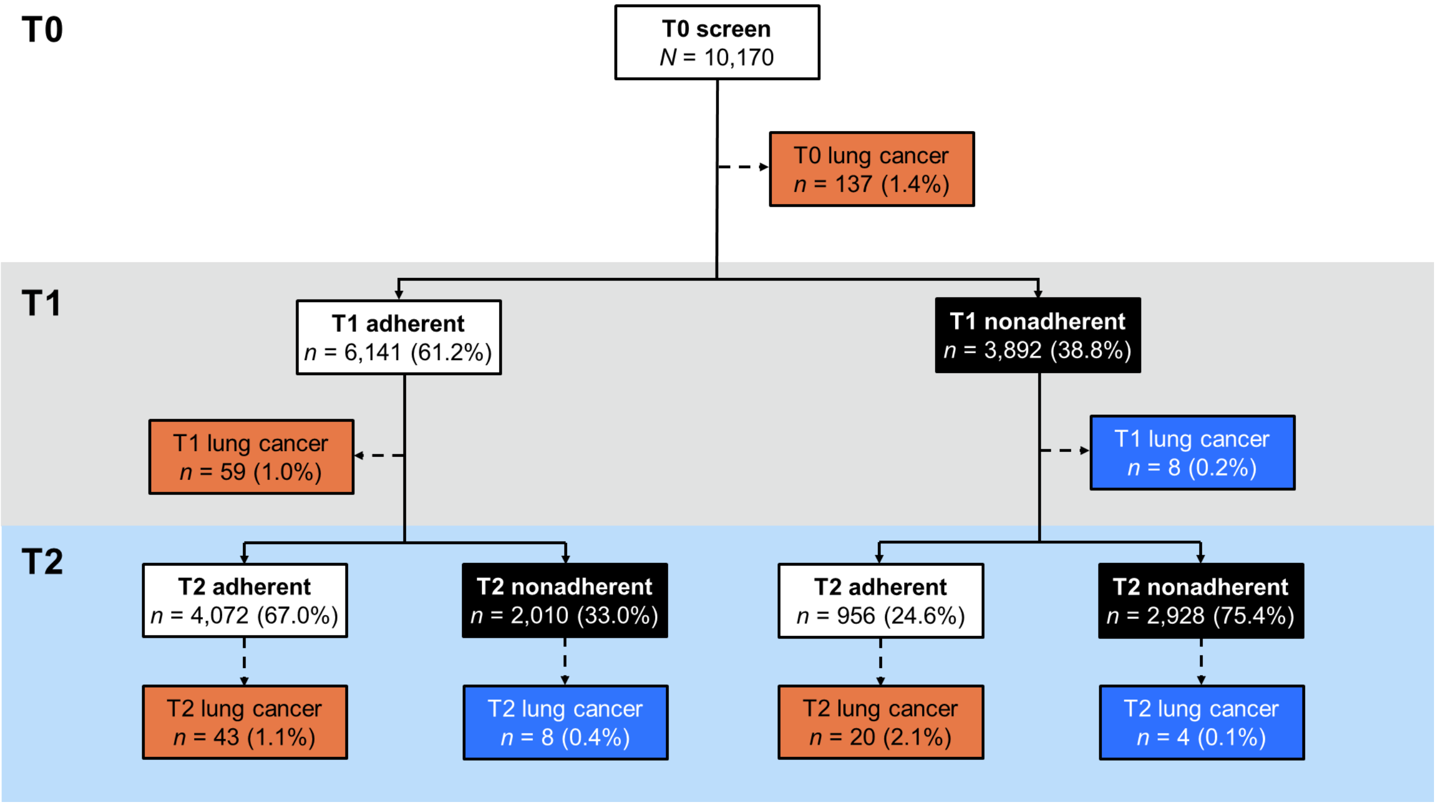

White boxes indicate individuals adherent to lung cancer screening, black boxes individuals nonadherent to screening, orange boxes lung cancers detected among individuals adherent to screening, and blue boxes lung cancers detected among individuals nonadherent to screening.

© 2025 Kim RY et al. *JAMA Network Open*.

eReferences.

1. Rendle KA, Burnett-Hartman AN, Neslund-Dudas C, et al. Evaluating Lung Cancer Screening Across Diverse Healthcare Systems: A Process Model from the Lung PROSPR Consortium. *Cancer Prev Res (Phila).* 2020;13(2):129-136.
2. von Elm E, Altman DG, Egger M, et al. The Strengthening the Reporting of Observational Studies in Epidemiology (STROBE) statement: guidelines for reporting observational studies. *Ann Intern Med.* 2007;147(8):573-577.
3. Quan H, Li B, Couris CM, et al. Updating and validating the Charlson comorbidity index and score for risk adjustment in hospital discharge abstracts using data from 6 countries. *Am J Epidemiol.* 2011;173(6):676-682.
4. Yu M, Tatalovich Z, Gibson JT, Cronin KA. Using a composite index of socioeconomic status to investigate health disparities while protecting the confidentiality of cancer registry data. *Cancer Causes Control.* 2014;25(1):81-92.
5. Flanagin A, Frey T, Christiansen SL, Bauchner H. The Reporting of Race and Ethnicity in Medical and Science Journals: Comments Invited. *JAMA.* 2021;325(11):1049-1052.
6. American College of Radiology. Lung CT Screening Reporting and Data System (Lung-RADS). https://www.acr.org/Clinical-Resources/Reporting-and-Data-Systems/Lung-Rads. Accessed November 27, 2024.
7. Kim RY, Rendle KA, Mitra N, et al. Racial Disparities in Adherence to Annual Lung Cancer Screening and Recommended Follow-Up Care: A Multicenter Cohort Study. *Ann Am Thorac Soc.* 2022;19(9):1561-1569.
8. Kim RY, Rendle KA, Mitra N, et al. Socioeconomic Status as a Mediator of Racial Disparity in Annual Lung Cancer Screening Adherence. *Am J Respir Crit Care Med.* 2023;207(6):777-780.
9. Rendle KA, Saia CA, Vachani A, et al. Rates of Downstream Procedures and Complications Associated With Lung Cancer Screening in Routine Clinical Practice : A Retrospective Cohort Study. *Ann Intern Med.* 2024;177(1):18-28.
10. Zou G. A modified poisson regression approach to prospective studies with binary data. *Am J Epidemiol.* 2004;159(7):702-706.
11. McNutt LA, Wu C, Xue X, Hafner JP. Estimating the relative risk in cohort studies and clinical trials of common outcomes. *Am J Epidemiol.* 2003;157(10):940-943.
12. White IR, Royston P, Wood AM. Multiple imputation using chained equations: Issues and guidance for practice. *Stat Med.* 2011;30(4):377-399.
13. van Buuren S. Multiple imputation of discrete and continuous data by fully conditional specification. *Stat Methods Med Res.* 2007;16(3):219-242.

© 2025 Kim RY et al. *JAMA Network Open*.
