# Supplementary material for: Adherence to Annual Lung Cancer Screening and Rates of Cancer Diagnosis
Source: JAMA Netw Open. Author manuscript; Available in PMC 2025 Apr 12. (PMC11920840; doi:10.1001/jamanetworkopen.2025.0942)
Supplement: Supplement 2 — Data Sharing Statement [file NIHMS2067533-supplement-Supplement_2.pdf]

Data Sharing Statement

Kim. Adherence to Annual Lung Cancer Screening and Rates of Cancer Diagnosis. *JAMA Netw Open*. Published March 18, 2025. doi:10.1001/jamanetworkopen.2025.0942

Data
Data available: No

Additional Information
Explanation for why data not available: The analytic data set is available to approved individuals with written approvals from PROSPR-Lung consortium sites
